# DCE‐MRI reveals reduced BBB permeability in basal forebrain in AD patients

**DOI:** 10.1002/alz70856_102261

**Published:** 2025-12-25

**Authors:** Ondrej Lerch, David Kala, Zuzana Nedelska, Haris Hadzic, Jakub Otahal, Jakub Hort

**Affiliations:** ^1^ Charles University, Second Faculty of Medicine and Motol University Hospital, Prague, Czech Republic

## Abstract

**Background:**

Blood brain barrier (BBB) dysfunctions is one of is one of the possible mechanisms contributing to onset of Alzheimer's disease (AD), a progressive neurodegenerative disease and a leading cause of dementia worldwide. Dynamic contrast enhanced magnetic resonance imaging (DCE‐MRI) is an imaging technique allowing regional assessment of BBB breakdown by estimating local metrics of capillary permeability such as K‐trans (volume transfer constant). We used DCE‐MRI to examine BBB dysfunction in regions affected early in the course of AD ‐ hippocampus, entorhinal cortex (EC) and basal forebrain (BF) nuclei.

**Method:**

A group of 43 participants – 22 biomarker negative cognitively unimpaired (CU) individuals and 21 biomarker positive patients with mild cognitive impairment or dementia due to AD from Czech Brain Aging Study underwent DCE‐MRI. K‐trans maps were estimated using Patlak algorithm implemented within ROCKETSHIP software toolbox. Segmentations of hippocampal head, body and tail, anterolateral and posteromedial EC and BF nuclei were obtained using in house developed pipeline. Average K‐trans values were extracted for each region. Regional differences in BBB permeability between groups were assessed using ANCOVA adjusted for age, sex and ApoE4 positivity.

**Result:**

Participants in the AD group had lower mean K‐trans in total BF (K‐trans_CU_= 0.573 x10^‐3^min^‐1^; K‐trans_AD_= 0.270 x10^‐3^min^‐1^, *p* =  0.002), anterior‐intermediate (K‐trans_CU_= 0.833 x10^‐3^min^‐1^; K‐trans_AD_= 0.291 x10^‐3^min^‐1^, *p* =  0.002) and posterior (K‐trans_CU_ = 0.536 x10^‐3^min^‐1^; K‐trans_AD_ = 0.237 x10^‐3^min^‐1^, *p* =  0.004) part of nucleus basalis Meynerti. There were no other significant differences between regional BBB permeability in measured structures (*p* >0.05)

**Conclusion:**

Our data show regional reduction in BBB permeability BF region but not other regions in patients with MCI due to AD and AD dementia. Regional BBB dysfunction may be one of the factors contributing to early AD related pathological changes in the BF area.

